# Influence of Demographic Characteristics on Trait Emotional Intelligence and Treatment Adherence Among Patients on Hemodialysis

**DOI:** 10.7759/cureus.96368

**Published:** 2025-11-08

**Authors:** Orchan Impis, Afroditi Zartaloudi, Eirini Grapsa, Georgia Gerogianni

**Affiliations:** 1 Department of Nursing, University of West Attica, Athens, GRC; 2 Department of Nephrology, Aretaeio Hospital, Athens, GRC

**Keywords:** demographic characteristics, emotional intelligence, hemodialysis patients, treatment adherence, well-being

## Abstract

Introduction

Trait emotional intelligence refers to individuals’ capability to precisely identify and interpret emotions, use emotional information to support cognitive processes, and regulate emotional states effectively. The aim of the study was to explore the influence of demographic characteristics on emotional intelligence and treatment adherence in individuals undergoing hemodialysis.

Methods

In this study, a total of 468 patients on hemodialysis completed these questionnaires: i) Trait Emotional Intelligence Questionnaire Short Form (TEIQue-SF), ii) GR-Simplified Medication Adherence Questionnaire-Hemodialysis (GR-SMAQ-HD), and iii) A questionnaire about demographic and clinical characteristics. Spearman’s correlation coefficients were utilized to investigate the association between two continuous variables. Multiple linear regression analysis was utilized with the dependent variables, the dimensions of GR-SMAQ-HD and TEIQue-SF scale.

Results

Kidney transplant candidates had high overall emotional intelligence (p=0.007), while patients who were women demonstrated low overall emotional intelligence (p=0.005). Patients with a fistula or graft demonstrated enhanced self-control (p=0.022, p=0.026, respectively) and increased well-being (p=0.024). Older age was associated with high overall treatment adherence (p<0.001), while patients living alone and those with comorbidities demonstrated low levels of overall treatment adherence (p=0.007 and p=0.049, respectively).

Conclusions

Educational and psychosocial programs designed to improve emotional intelligence can be incorporated into hemodialysis care, in order to foster greater treatment adherence among patients on hemodialysis.

## Introduction

Emotional intelligence as a trait refers to individuals' self-perceptions of their emotional world, including their perceived abilities to understand, express, and regulate emotions, in order to adapt to the environment and sustain well-being [1]. Trait emotional intelligence integrates the emotional aspects of the personality into a comprehensive model, consisting of 15 dimensions, which are grouped into four broad domains: (i) self-control, (ii) well-being, (iii) emotionality, and (iv) sociability [2]. 

Patients undergoing hemodialysis constitute a distinct population category in terms of treatment compliance, due to the complexity of the treatment, which has a significant impact on individuals’ daily lives [3]. Treatment adherence refers to patients’ compliance with medication, and adherence to dialysis sessions and diet and fluid restrictions [4]. Patient non-compliance with hemodialysis is a common problem among this population, especially in individuals who have been undergoing dialysis for a long time. Non-adherence to treatment usually leads to cardiovascular mortality or premature death [5].

Research has demonstrated a link between emotional intelligence and treatment adherence in the population undergoing dialysis, with the potential influence of sociodemographic factors being increasingly recognized. In a cross-sectional study involving 80 kidney transplant recipients, emotional intelligence was significantly associated with treatment compliance [6], while in a similar study involving 218 patients undergoing hemodialysis, it was found that increased age was associated with higher treatment adherence among these individuals [7]. Although a variety of studies have investigated emotional intelligence or treatment adherence among patients on hemodialysis in Greece, few have explored the influence of demographic characteristics on trait emotional intelligence and treatment adherence in the Greek population.

The aim of this study was to explore how demographic variables influence trait emotional intelligence and treatment adherence among patients on hemodialysis.

## Materials and methods

Study sample

Participants fulfilled the requirements for participation if they were between 18 and 85 years of age, had been receiving hemodialysis treatment for a minimum of three months, and possessed adequate proficiency in reading, writing, and speaking the Greek language. Exclusion criteria included being older than 85 years, limited ability to read, write, or speak Greek, a history of alcohol or substance abuse, and the presence of psychiatric disorders or cognitive impairment. Cognitive impairment was assessed based on the medical record and the clinical evaluation of the medical and nursing team. The study took place between November 2023 and July 2024.

A total of 887 patients who met the inclusion criteria were invited to participate in the study. Of these, 80 declined participation during the initial recruitment phase and were therefore excluded. Consequently, 807 patients were approached for data collection in the dialysis units. Among them, 187 patients (23.17%) did not ultimately take part, mainly due to temporary health problems or withdrawal before the data collection began. As a result, 620 patients accepted to participate (response rate: 76.85%) and completed the questionnaires. Of these, 468 (57.99%) participants provided fully-completed questionnaires, while 152 (18.83%) provided partially-completed questionnaires, due to fatigue or limited concentration during the dialysis session. The latter were excluded from the final analysis due to incomplete data.

The data were collected through face-to-face interactions in dialysis units, where patients completed the questionnaires themselves with the researcher present to clarify any questions. Participants completed the following questionnaires: (i) Trait Emotional Intelligence Questionnaire-short form (TEIQue-SF), (ii) GR-Simplified Medication Adherence Questionnaire-Hemodialysis (GR-SMAQ-HD), and (iii) a questionnaire about demographic and clinical characteristics.

It was calculated that with the sample size of 400 participants or more, the study will have 99% power to perform a linear regression analysis with dependent variables of the study outcomes, at a significance level of 0.05, and for effect sizes equal to or greater to 0.15.

Instruments

Trait Emotional Intelligence Questionnaire Short Form (TEIQue-SF)

This has been created for the evaluation of overall emotional intelligence as a trait [8] and constitutes a short version of the full questionnaire TEIQue [9]. The short form has 30 items and responses are assessed using a seven-point Likert scale [8], where elevated scores correspond to greater emotional intelligence. A Greek version of the questionnaire was developed through translation [10] and is available free of charge for academic use from the London Psychometric Laboratory. The TEIQue-SF exhibits strong validity and reliability within the Greek population [11]. 

GR-Simplified Medication Adherence Questionnaire-Hemodialysis (GR-SMAQ-HD)

This is a modified instrument for assessing the adherence of patients on hemodialysis and evaluates all dimensions of compliance to treatment. This questionnaire comprises eight questions which are divided into three subscales: (i) compliance with medication regimen, (ii) adherence to hemodialysis treatments, and (iii) dietary intake/fluid restriction. The total score varies from zero to eight, with higher scores reflecting higher compliance. The questionnaire exhibited satisfactory internal consistency and validity within the Greek population [12]. Permission to use the GR-SMAQ-HD scale was obtained for this study.

Statistical analysis

Quantitative variables were presented as as mean values (SD) or as median (IQR), while qualitative variables were presented as absolute and relative frequencies. Spearman’s correlation coefficients were utilized to investigate the relation of the two continuous variables [13].

Multiple linear regression analysis was conducted using the overall treatment adherence score (GR-SMAQ-HD) as the dependent variable. In the first step, respondents’ demographic and clinical characteristics were entered. In the second step subscales of emotional intelligence were entered in a stepwise method (p for entry 0.05, p for removal 0.10). Multiple linear regression analyses were also used with dependent variables being the dimensions of the TEIQue-SF scale and independent participants’ demographic and clinical characteristics. Adjusted regression coefficients (β) with standard errors (SE) were computed from the results of the linear regression analyses. Log transformations were utilized for the dependent variable due to lack of normal distribution. All reported p values were two-tailed. Statistical significance was set at p<0.05 and statistical analyses were carried out with IBM SPSS Statistics for Windows, Version 26 (Released 2019; IBM Corp., Armonk, New York, United States) [13,14].

Ethics

The study’s objectives and procedures were explained to participants, and they were informed about the confidentiality of their data. Prior to data collection, approval was obtained from the Ethics Committee of the University of West Attica (Approval Number: 100757/24-10-2023). The study was carried out in compliance with the Declaration of Helsinki (1989).

## Results

Characteristics of participants

The study sample included 468 patients undergoing hemodialysis, with a mean age of 63.9 years (SD=13.1). Males represented 61.3% of the study, and the majority (96.4%) were citizens of Greece. Most participants (75.4%) lived in the wider Attica area. Regarding level of academic education, 43.6% had completed secondary education (middle or high school), while 28.2% held degrees from universities or technological institutes. The majority were married (65.6%) and had children (81.8%), with a median of two children among those who were parents. In addition, 20.1% reported living alone, while most of them were retired (69.7%). The median period of time they had been receiving hemodialysis treatment was 36 months (IQR: 16-72 months), with 95.5% of participants undergoing dialysis sessions three times per week. Each session lasted an average of 3.8 hours (SD=0.4).

Vascular access included an arteriovenous fistula in 65.4% of cases, a central venous catheter in 24.2%, and a graft in 9.4% of the participants. Additionally, 38.5% of the respondents were on a waiting list for kidney transplantation, and 7.9% had previously received a kidney transplantation. Finally, comorbidities were present in 73.7% of the sample (Table 1).

**Table 1:** Characteristics of the participants

Demographic variables			Value
Sex, N (%)	Male		287 (61.3%)
	Female		179 (38.2%)
	Other		2 (0.4%)
Age, mean (years) (SD)			63.9 (13.1)
Ethnic origin, N (%)	Greek		451 (96.4%)
	Other		17 (3.6%)
Living in the wider Attica area, N (%)	No		115 (24.6%)
	Yes		353 (75.4%)
Level of education, N (%)	Primary school		91 (19.4%)
	Middle school - High school		204 (43.6%)
	Technological Educational Institute / University		132 (28.2%)
	Master's degree - PhD		41 (8.8%)
Married or cohabiting, N (%)	No		161 (34.4%)
	Yes		307 (65.6%)
Presence of children, N (%)	No		85 (18,2%)
	Yes		383 (81,8%)
Living alone, N (%)	No		374 (79,9%)
	Yes		94 (20.1%)
Employment status, N (%)	Public servant		14 (3%)
	Self-employed		30 (6.4%)
	Unemployed		21 (4.5%)
	Private-sector employee		43 (9.2%)
	Household		32 (6.8%)
	Student		2 (0.4%)
	Pensioner		326 (69.7%)
Financial status, N (%)	Poor		42 (9%)
	Moderate		217 (46.4%)
	Good		167 (35.7%)
	Very good		35 (7.5%)
	Excellent		7 (1.5%)
Months of hemodialysis, Median (IQR)			36.0 (16-72)
Session duration (hours), Mean (SD)			3.8 (0.4)
Type of vascular access, N (%)		Fistula	306 (65.4%)
		Graft	44 (9.4%)
		Catheter	114 (24.2%)
Health insurance, N (%)		Public	403 (86.1%)
		Private	17 (3.6%)
		Both	35 (7.5%)
		Uninsured	13 (2.8%)
Are you candidate for kidney transplantation? N (%)		No	288 (61.5%)
		Yes	180 (38.5%)
Have you had a kidney transplantation in the past? N (%)		No	431 (92.1%)
		Yes	37 (7.9%)
Comorbid diseases, N (%)		No	124 (26.3%)
		Yes	345 (73.7%)

Descriptive statistics for TEIQue-SF and GR-SMAQ-HD

Concerning the TEIQue-SF scale, the mean score for the "Emotionality" dimension was 4.8 (SD=0.9), "Self-control" dimension 4.6 (SD=1.0), "Well-being" dimension 4.8 (SD=1.2), and "Sociability" dimension 4.3 (SD=1.0). The overall mean score was 4.7 (SD=0.8). Higher values indicate greater emotionality, self-control, well-being, sociability, and overall Trait emotional intelligence, respectively. Regarding GR-SMAQ-HD scale, the overall mean score for treatment adherence was 6.6 (SD=1.5), where higher values indicate better overall treatment adherence (Table 2).

**Table 2:** Descriptive statistics for TEIQue-SF and GR-SMAQ-HD scales TEIQue-SF: Trait Emotional Intelligence Questionnaire Short Form; GR-SMAQ-HD: GR-Simplified Medication Adherence Questionnaire-Hemodialysis; IQR: Interquartile range.

	Minimum	Maximum	Mean (SD)	Median (IQR)
GR-SMAQ-HD				
Total Compliance Scale	1.0	8.0	6.6 (1.5)	7 (6-8)
Compliance with medication regimen	0.0	4.0	3.2 (1)	4 (2-4)
Adherence to hemodialysis treatments	0.0	2.0	1.9 (0.4)	2 (2-2)
Dietary intake /fluid restriction	0.0	2.0	1.5 (0.7)	2 (1-2)
TEIQue-SF				
Emotionally	2.0	6.5	4.8 (0.9)	4.8 (4.1-5.5)
Self-control	2.3	6.8	4.6 (1)	4.5 (4-5.3)
Well-being	2.0	7.0	4.8 (1.2)	4.8 (4-5.7)
Sociability	1.7	6.8	4.3 (1)	4.3 (3.7-4.9)
Total TEIQue score	2.8	6.4	4.7 (0.8)	4.6 (4.1-5.3)

Spearman’s correlation coefficients between TEIQue-SF and GR-SMAQ-HD scales

The dimensions of self-control, well-being, and the overall TEIQue-SF score were positively associated with better compliance with medication regimen (p<0.05, p<0.001, and p<0.05, respectively), better adherence to hemodialysis treatments (p<0.001, p<0.01, p<0.05, respectively), and better overall compliance (p<0.01, p<0.001, p<0.001, respectively). Increased well-being and overall emotional intelligence were also associated with greater adherence to fluid and dietary restrictions (p<0.01) (Table 3).

**Table 3:** Spearman’s correlation coefficients between GR-SMAQ-HD’s and TEIQue-SF’s dimensions *p<0.05; **p<0.01; ***p<0.001; TEIQue-SF: Trait Emotional Intelligence Questionnaire Short Form; GR-SMAQ-HD: GR-Simplified Medication Adherence Questionnaire-Hemodialysis.

		GR-SMAQ-HD			
		Total compliance scale	Compliance with medication regimen	Adherence to hemodialysis treatments	Dietary intake/fluid restriction compliance
TEIQue-SF	Emotionally	0.06	0.02	-0.02	0.09
	Self-control	0.14**	0.11*	0.17***	0.04
	Well being	0.24***	0.18***	0.14**	0.14**
	Sociability	0.02	-0.02	0.05	0.03
	Total TEIQue-SF score	0.17***	0.11*	0.12*	0.12**

Multiple linear regression of factors independently associated with emotional intelligence dimensions (TEIQue-SF)

To identify the factors independently associated with the dimensions of emotional intelligence, multiple linear regression analyses were carried out. The dependent variables were the scores on each dimension of emotional intelligence, while the independent variables included the demographic and clinical characteristics of respondents.

Individuals who had undergone a kidney transplant in the past had a higher score in the emotionality dimension compared to those who had not (β=0.039, p=0.015). Additionally, women exhibited lower levels of self-control than men (β=-0.029, p=0.002), while patients with a fistula (β=0.025, p=0.022) or graft (β=0.038, p=0.026) exhibited better self-control compared to those with a central venous catheter. Also, kidney transplant candidates showed greater self-control than non-candidates (β=0.026, p=0.011).

Moreover, women reported lower well-being scores compared to men (β=-0.028, p=0.014), while higher satisfaction with one’s financial situation was linked to increased well-being (β=0.029, p<0.001). Furthermore, patients with a fistula reported greater well-being than those using a central venous catheter (β=-0.030, p=0.024), while kidney transplant candidates showed higher well-being scores in comparison to those not on the transplant waiting list (β=-0.034, p=0.008). 

In addition, women exhibited lower sociability than men (β=-0.021, p=0.043), while employed participants demonstrated greater sociability compared to those who were unemployed (β=0.037, p=0.011).

Finally, gender, satisfaction with financial status, and kidney transplant candidacy were found to be associated with overall emotional intelligence, as evaluated with the TEIQue-SF global score. Specifically, women exhibited lower overall emotional intelligence levels compared to men (β=-0.020, p=0.005). Greater satisfaction with one’s financial situation was linked to higher overall emotional intelligence (β=0.011, p=0.008), while individuals who were candidates for kidney transplantation demonstrated higher overall emotional intelligence scores than those who were not on the transplant list (β=0.021, p=0.007) (Tables 4, 5). 

**Table 4:** Multiple linear regression of factors independently related to emotional intelligence dimensions (TEIQue-SF) Logarithmic transformation of the dependent variable was used for this analysis; TEIQue-SF: Trait Emotional Intelligence Questionnaire Short Form; ^+^regression coefficient;  ^++^Standard Error; ^a^F(17,444)=1.75; p=0.033; R2=0.06; R2adj=0.03; ^b^F(17,444)=2.68; p<0.001; R2=0.09; R2adj=0.06; ^c^F(17,444)=3.39; p<0.001; R2=0.12; R2adj=0.08; ^d^F(17,444)=1.90; p=0.016; R2=0.07; R2adj=0.03.

	Emotionally^a^		Self-control^b^		Well being^c^		Sociability^d^		
	β^+^ (95% CI^++^)	P	β^+^ (95% CI^++^)	P	β^+^ (95% CI^++^)	P		β^+^ (95% CI^++^)	P
Sex (Female vs Male)	-0.003 (-0.021 ─ 0.014)	0.715	-0.029 (-0.047 ─ -0.010)	0.002	-0.028 (-0.051 ─ -0.006)	0.014		-0.021 (-0.042 ─ -0.001)	0.043
Age	0.000 (-0.001 ─ 0.001)	0.701	0.001 (0.000 ─ 0.002)	0.085	0.001 (-0.001 ─ 0.002)	0.371		0.001 (-0.001 ─ 0.002)	0.323
Living in the wider Attica area (Yes vs No)	0.000 (-0.020 ─ 0.019)	0.977	-0.003 (-0.024 ─ 0.017)	0.735	0.009 (-0.016 ─ 0.034)	0.473		-0.016 (-0.038 ─ 0.007)	0.177
Level of education: Middle school - High school vs Primary school	0.007 (-0.016 ─ 0.031)	0.545	0.010 (-0.015 ─ 0.034)	0.433	-0.004 (-0.035 ─ 0.026)	0.776		0.013 (-0.015 ─ 0.041)	0.365
Level of education: Technological Educational Institute / University vs Primary school	0.025 (-0.001 ─ 0.052)	0.056	0.023 (-0.004 ─ 0.05)	0.101	0.002 (-0.032 ─ 0.036)	0.905		0.023 (-0.007 ─ 0.054)	0.137
Level of education: Master's degree - PhD vs Primary school	0.024 (-0.012 ─ 0.061)	0.194	0.030 (-0.008 ─ 0.068)	0.118	-0.016 (-0.064 ─ 0.031)	0.495		0.026 (-0.017 ─ 0.069)	0.240
Married or cohabiting (Yes vs No)	-0.015 (-0.037 ─ 0.007)	0.190	0.000 (-0.023 ─ 0.024)	0.972	-0.032 (-0.061 ─ 0.003)	0.061		0.015 (-0.011 ─ 0.042)	0.264
Presence of children (Yes vs No)	0.006 (-0.018 ─ 0.031)	0.598	-0.003 (-0.028 ─ 0.022)	0.792	0.018 (-0.013 ─ 0.049)	0.249		-0.012 (-0.040 ─ 0.017)	0.424
Living alone (Yes vs No)	-0.002 (-0.027 ─ 0.024)	0.903	0.009 (-0.018 ─ 0.036)	0.517	-0.023 (-0.056 ─ 0.010)	0.173		0.016 (-0.014 ─ 0.046)	0.294
Employed (Yes vs No)	-0.009 (-0.033 ─ 0.016)	0.479	0.000 (-0.026 ─ 0.025)	0.991	-0.003 (-0.035 ─ 0.028)	0.829		0.037 (0.009 ─ 0.066)	0.011
Financial status	0.001 (-0.010 ─ 0.011)	0.899	0.005 (-0.006 ─ 0.016)	0.367	0.029 (0.016 ─ 0.043)	<0.001		0.009 (-0.003 ─ 0.022)	0.140
Months of hemodialysis	0.013 (-0.008 ─ 0.034)	0.238	0.016 (-0.006 ─ 0.038)	0.157	-0.003 (-0.031 ─ 0.024)	0.81		0.017 (-0.008 ─ 0.042)	0.182
Type of vascular access: Fistula vs Catheter	-0.005 (-0.025 ─ 0.016)	0.656	0.025 (0.004 ─ 0.046)	0.022	0.030 (0.004 ─ 0.056)	0.024		-0.008 (-0.032 ─ 0.015)	0.484
Type of vascular access: Graft vs Catheter	0.010 (-0.021 ─ 0.042)	0.519	0.038 (0.005 ─ 0.071)	0.026	0.037 (-0.004 ─ 0.078)	0.078		-0.004 (-0.041 ─ 0.033)	0.834
Comorbid diseases (Yes vs No)	0.003 (-0.017 ─ 0.022)	0.773	0.007 (-0.014 ─ 0.027)	0.510	-0.020 (-0.045 ─ 0.006)	0.128		0.000 (-0.023 ─ 0.023)	0.985
Are you candidate for kidney transplantation? (Yes vs No)	0.018 (-0.002 ─ 0.037)	0.074	0.026 (0.006 ─ 0.047)	0.011	0.034 (0.009 ─ 0.059)	0.008		0.008 (-0.015 ─ 0.031)	0.501
Have you had a kidney transplantation in the past? (Yes vs No)	0.039 (0.008 ─ 0.070)	0.015	0.006 (-0.026 ─ 0.039)	0.712	0.004 (-0.037 ─ 0.044)	0.86		0.008 (-0.029 ─ 0.045)	0.669

**Table 5:** Multiple linear regression of factors independently associated with total TEIQue-SF score TEIQue-SF: Trait Emotional Intelligence Questionnaire Short Form; ^+^regression coefficient; ^++^Standard Error; Logarithmic transformation of the dependent variable was used for this analysis;  F(17,444)=3.37; p<0.001; R2=0.11; R2adj=0.08.

	Total TEIQue-SF score	
	β^+^ (95% CI^++^)	P
Sex (Female vs Male)	-0.020 (-0.034 ─ -0.006)	0.005
Age	0.000 (-0.001 ─ 0.001)	0.651
Living in the wider Attica area (Yes vs No)	-0.003 (-0.018 ─ 0.012)	0.690
Level of education: Middle school - High school vs Primary school	0.005 (-0.014 ─ 0.024)	0.586
Level of education: Technological Educational Institute / University vs Primary school	0.019 (-0.002 ─ 0.039)	0.080
Level of education: Master's degree - PhD vs Primary school	0.019 (-0.010 ─ 0.048)	0.197
Married or cohabiting (Yes vs No)	-0.010 (-0.027 ─ 0.008)	0.296
Presence of children (Yes vs No)	0.005 (-0.015 ─ 0.024)	0.631
Living alone (Yes vs No)	-0.002 (-0.023 ─ 0.018)	0.841
Employed (Yes vs No)	0.001 (-0.018 ─ 0.021)	0.883
Financial status	0.011 (0.003 ─ 0.020)	0.008
Months of hemodialysis	0.009 (-0.008 ─ 0.026)	0.292
Type of vascular access: Fistula vs Catheter	0.011 (-0.005 ─ 0.027)	0.170
Type of vascular access: Graft vs Catheter	0.020 (-0.006 ─ 0.045)	0.127
Comorbid diseases (Yes vs No)	-0.004 (-0.019 ─ 0.012)	0.625
Are you candidate for kidney transplantation? (Yes vs No)	0.021 (0.006 ─ 0.037)	0.007
Have you had a kidney transplantation in the past? (Yes vs No)	0.016 (-0.009 ─ 0.041)	0.211

Multiple linear regression of factors independently associated with adherence to treatment (GR-SMAQ-HD)

To identify the factors independently associated with overall patient treatment adherence, a multiple linear regression analysis was carried out. The independent variables included participants’ demographic, clinical characteristics and emotional intelligence (TEIQue-SF). A hierarchical regression approach was used; in the first step, demographic and clinical variables were introduced with the enter method, while in the second step, the dimensions of the emotional intelligence were introduced.

Older age was associated with higher overall treatment adherence (β=0.002, p<0.001), while patients living alone (β=-0.039, p=0.007) demonstrated lower levels of overall treatment adherence compared to those living with others. Also, significantly lower overall treatment adherence was found in those with comorbidities (β=-0.021, p=0.049). Finally, increased well-being was associated with greater total adherence (β=0.019, p<0.001). (Table 6).

**Table 6:** Multiple linear regression of factors independently associated with adherence to treatment (GR-SMAQ-HD) GR-SMAQ-HD: GR-Simplified Medication Adherence Questionnaire-Hemodialysis​​​​​​; ^+^regression coefficient; ^++^Standard Error; Logarithmic transformation of the dependent variable was used for this analysis; F(18,444)=4.41; p<0.001; R2=0.15; R2adj=0.12.

	Total GR-SMAQ-HD	
	β^+^ (95% CI^++^)	P
Sex (Female vs Male)	0.005 (-0.015 ─ 0.024)	0.621
Age	0.002 (0.001 ─ 0.003)	<0.001
Living in the wider Attica area (Yes vs No)	-0.020 (-0.041 ─ 0.002)	0.074
Level of education: Middle school - High school vs Primary school	0.012 (-0.015 ─ 0.038)	0.380
Level of education: Technological Educational Institute / University vs Primary school	0.004 (-0.026 ─ 0.033)	0.807
Level of education: Master's degree - PhD vs Primary school	-0.019 (-0.059 ─ 0.022)	0.367
Married or cohabiting (Yes vs No)	-0.009 (-0.034 ─ 0.016)	0.491
Presence of children (Yes vs No)	-0.01 (-0.037 ─ 0.017)	0.460
Living alone (Yes vs No)	-0.039 (-0.068 ─ -0.011)	0.007
Employed (Yes vs No)	-0.007 (-0.035 ─ 0.020)	0.591
Financial status	0.005 (-0.007 ─ 0.017)	0.381
Months of hemodialysis	-0.012 (-0.035 ─ 0.012)	0.324
Type of vascular access: Fistula vs Catheter	0.009 (-0.014 ─ 0.031)	0.445
Type of vascular access: Graft vs Catheter	-0.010 (-0.046 ─ 0.025)	0.563
Comorbid diseases (Yes vs No)	-0.021 (-0.043 ─ 0.000)	0.049
Are you candidate for kidney transplantation? (Yes vs No)	0.017 (-0.005 ─ 0.039)	0.129
Have you had a kidney transplantation in the past? (Yes vs No)	0.026 (-0.008 ─ 0.061)	0.137
Well-being	0.019 (0.011 ─ 0.027)	<0.001

## Discussion

The findings of this study indicated that kidney transplant candidates had higher well-being (p=0.008), greater self-control (p=0.011), and higher overall emotional intelligence (p=0.007) compared to those not on the transplant waiting list. This may be attributed to patients’ expectation that transplantation will result in both extended longevity and enhanced quality of life [15]. More specifically, the prospect of transplantation provides patients with hope for overcoming the limitations imposed by their illness and returning to daily routines, while also motivating them to actively manage their condition. It is important to note that social support from family and healthcare professionals during the waiting period for transplantation has a positive effect on patients’ mental and physical well-being [16]. These findings are consistent with a similar study of 1,838 patients undergoing dialysis, which found that individuals on the kidney transplant waiting list reported fewer symptoms of depression and higher quality of life than those not on the waiting list [17].

Furthermore, individuals who had undergone a kidney transplant in the past had higher emotionality compared to those who had not (p=0.015). This is probably influenced by improvements in health following kidney transplantation, such as enhanced quality of life, a return to normality, increased energy, and an increased sense of appreciation and meaning in life [18]. This finding is consistent with those of a similar study involving 100 individuals who had undergone kidney transplantation and 272 patients on hemodialysis. The results showed that patients who had undergone a kidney transplant demonstrated better quality of life, particularly in the sub-dimensions of emotional role, mental health and social functioning, in comparison with patients who were still undergoing hemodialysis [19].

Moreover, the results of this study found that employed participants demonstrated greater sociability compared to those who were unemployed (p=0.011). It can be assumed that a work environment can provide opportunities for social relationships and interaction among people with chronic diseases [20], while it allows individuals to socialize with others, fostering a sense of personal worth, and reinforcing the feeling that they maintain a social role and contribute to society [21]. In a similar study of 441 patients undergoing dialysis, it was found that those who were employed demonstrated higher levels of participation in social activities compared to their unemployed counterparts [22].

Additionally, this study found that patients with a fistula or graft demonstrated better self-control (p=0.022 and p=0.026, respectively) and greater well-being (p=0.024), which are dimensions of trait emotional intelligence, compared to those using a central venous catheter for hemodialysis. This can be attributed to the fact that patients with an arteriovenous fistula have increased health perception, higher vigor, and greater satisfaction with the type of vascular access [23], since they have fewer restrictions on physical activity [24], and low incidence of complications and mortality [25]. In contrast, central venous catheters are frequently associated with infections and complications such as central vein stenosis [26], frequent hospitalizations [27], and high morbidity, and patients are often concerned about their appearance [24]. Similar findings were reported in a study of 1,461 dialysis patients, which found that those with a arteriovenous fistula or graft had lower levels of depression and higher quality of life than individuals with a central venous catheter [28].

In addition, this study showed that women demonstrated lower levels of self-control (p=0.002), lower well-being scores (p=0.014), lower sociability (p=0.043), and lower overall emotional intelligence compared to men (p=0.005). This finding may be related to social and cultural factors associated with female gender, social stereotypes, and women’s access and utilization of resources [29] such as access to education and health services [30]. Also, women who experience illness usually continue to perform the roles of wife, mother, or daughter, putting others’ needs before their own [31]. Similar results were observed in a study of 70 patients undergoing renal replacement therapy, which found that men had higher mental quality of life compared to women [32].

Concerning adherence to treatment, this study showed that older age was associated with higher overall treatment adherence (p<0.001). This can be possibly explained by the fact that patients of an increased age tend to have a structured lifestyle and adapt easily to the demands of their treatment [33]. Similar results have been reported in previous studies involving patients undergoing dialysis, which found that older patients demonstrated higher levels of adherence to treatment compared to younger ones [34,35].

Additionally, the results of this study indicated that patients living alone and those with comorbidities demonstrated lower levels of overall treatment adherence (p=0.007 and p=0.049, respectively). Chronic kidney disease can lead to reduced self-confidence, increased feelings of loneliness, and increased dependence on family and health professionals [36]. Also, patients on dialysis often have a variety of comorbidities, leading to an increased number of medications, which have been reported as predictors of decreased adherence [37]. Also, lack of family and social support is often associated with the patient's non-compliance to the treatment regimen [38]. Similarly, in a study involving 170 patients undergoing hemodialysis, unmarried individuals were independently associated with a reduced compliance to fluid restrictions [39].

The findings of this study indicated that a variety of demographic and clinical characteristics were significantly associated with emotional intelligence and treatment adherence in patients undergoing hemodialysis. Thus, educational and psychosocial interventions aimed at enhancing emotional intelligence can be integrated into the care of such patients in order to promote better adherence behaviors. Such interventions may focus on emotion recognition and self-control, as well as patients’ understanding of their condition and strengthen self-management skills. Additionally, family involvement can further foster emotional resilience and motivation for treatment adherence among individuals on hemodialysis.

This study has certain limitations. The sample was drawn exclusively from dialysis units in the Attica region, possibly restricting the generalizability of the results to other geographical areas. Also, the possibility of non-response bias should be taken into consideration. The cross-sectional design is suitable for an initial investigation of the relationships between variables but inherently limits conclusions about causality. Future studies should address these theoretical and methodological limitations by incorporating mediation analyses, broader psychosocial variables, and careful control for potential confounding factors.

## Conclusions

A variety of demographic and clinical factors were found to be significantly associated with emotional intelligence and treatment adherence among patients undergoing hemodialysis. In this study, candidates for kidney transplant exhibited high overall emotional intelligence, while women demonstrated lower levels of overall emotional intelligence. Additionally, older age was associated with high overall treatment adherence, while patients living alone and those with comorbidities demonstrated lower levels of overall treatment adherence. These findings suggest that integrating emotion-focused interventions into routine dialysis care could potentially support emotional well-being and encourage better adherence to therapeutic regimens. 
